# Building Global Epidemiology and Response Capacity with Field Epidemiology Training Programs 

**DOI:** 10.3201/eid2313.170509

**Published:** 2017-12

**Authors:** Donna S. Jones, Richard C. Dicker, Robert E. Fontaine, Amy L. Boore, Jared O. Omolo, Rana J. Ashgar, Henry C. Baggett

**Affiliations:** US Centers for Disease Control and Prevention, Atlanta, Georgia, USA (D.S. Jones, R.C. Dicker, R.E. Fontaine, A.L. Boore, H.C. Baggett);; US Centers for Disease Control and Prevention, Kigali, Rwanda (J.O. Omolo);; Field Epidemiology and Laboratory Training Program, Islamabad, Pakistan (R.J. Ashgar)

**Keywords:** training, epidemiology, global health, global health security

## Abstract

More than ever, competent field epidemiologists are needed worldwide. As known, new, and resurgent communicable diseases increase their global impact, the International Health Regulations and the Global Health Security Agenda call for sufficient field epidemiologic capacity in every country to rapidly detect, respond to, and contain public health emergencies, thereby ensuring global health security. To build this capacity, for >35 years the US Centers for Disease Control and Prevention has worked with countries around the globe to develop Field Epidemiology Training Programs (FETPs). FETP trainees conduct surveillance activities and outbreak investigations in service to ministry of health programs to prevent and control infectious diseases of global health importance such as polio, cholera, tuberculosis, HIV/AIDS, malaria, and emerging zoonotic infectious diseases. FETP graduates often rise to positions of leadership to direct such programs. By training competent epidemiologists to manage public health events locally and support public health systems nationally, health security is enhanced globally.

In 1951, in response to the threat of biological warfare during the Korean War, the Communicable Disease Center (now the US Centers for Disease Control and Prevention; CDC) established the Epidemic Intelligence Service (EIS) to respond to infectious disease outbreaks (*1*). The 2-year training program used a learning-while-doing approach to develop field epidemiologists (or disease detectives) capable of rapidly investigating and curtailing public health threats. The EIS has served as the model for developing a similar program, called the Field Epidemiology Training Program (FETP), around the world (*2*).

In 1975, the first FETP outside the United States was established in Canada. In 1980, Thailand launched the first FETP outside of North America, with CDC support (*3*). Since then, FETPs have been established in ≈65 countries around the world, many with assistance from CDC, the World Health Organization (WHO), the European Centre for Disease Prevention and Control, and other public health organizations. In many countries, FETPs have proven to be successful models for building public health workforce capacity (*4*); however, critical gaps remain in epidemiologic capacity including, for example, the 3 countries in West Africa where the 2014–2015 Ebola epidemic arose and propagated widely (*5*).

In 2003, the outbreak of severe acute respiratory syndrome (*6*) highlighted the continued worldwide vulnerability to infectious disease threats brought by ever-expanding global travel and trade. In response to severe acute respiratory syndrome and similar threats, WHO revised the International Health Regulations in 2005 (IHR 2005) to define core capacities necessary for countries to detect and respond to public health threats (*7*). Unfortunately, many countries remain unprepared to meet IHR 2005 requirements. In 2014, the Global Health Security Agenda (GHSA) was launched by the United States with 28 partnering nations, WHO, the Food and Agriculture Organization, and the World Organisation for Animal Health. The GHSA purpose was to accelerate progress toward implementation of IHR 2005 so that all countries are able to rapidly detect, respond to, and control public health emergencies at their source and thereby ensure global health security (*6*). One of these core elements is adequate human resources, which is essential for achieving each of the other IHR 2005 capacities. Highlighting the role of workforce development in accelerated IHR 2005 implementation, WHO revised the IHR 2005 monitoring framework and the Joint External Evaluation tool (which is used to measure progress toward IHR 2005 and GHSA implementation) to include specific public health workforce targets that rely on having an “applied epidemiology training program in place such as FETP” (*8*). By 2014, however, nearly 70% of countries had still not achieved IHR 2005 compliance, and few countries had achieved the Joint External Evaluation target of having 1 trained field epidemiologist (or equivalent) per 200,000 population.

We describe the traditional 2-year FETP that has been supported by CDC in many countries. We also describe the effect of FETPs; their role in the development of a public health workforce; and how FETPs are enhancing the capacity of countries to rapidly detect, respond to, and control public health threats and thereby enhance global health security.

## Building Field Epidemiology Capacity Globally

**Figure 1:**
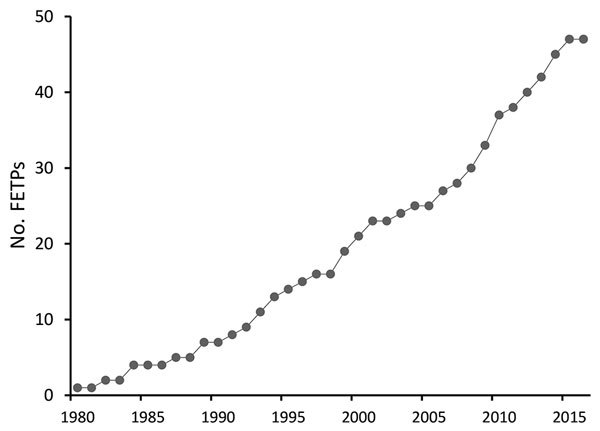
No. Field Epidemiology Training Programs (FETPs) established with US Centers for Disease Control and Prevention engagement (previous and current), 1980–2016.

**Figure 2:**
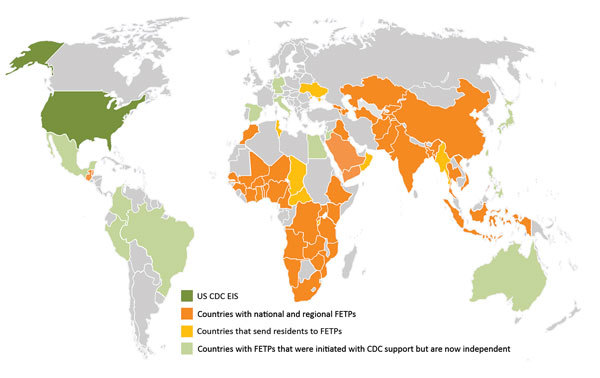
FETPs-Advanced presently or previously associated with CDC, as of December 2016. India supports 2 FETPs-Advanced; both were initiated with CDC support, and 1 is now independent. Central America has had an FETP-Advanced that was paused in 2015 and restarted in August 2017 with Guatemala and Belize. CDC, US Centers for Disease Control and Prevention; EIS, Epidemic Intelligence Service; FETP, Field Epidemiology Training Program.

CDC supports FETP development to strengthen countries’ epidemiology, surveillance, and response capacity, thereby enhancing global health security through a well-trained public health workforce. CDC support has included placement of a resident advisor in the country, technical support, and financial support. The resident advisor is an experienced applied epidemiologist, usually a graduate of the CDC EIS program or another FETP, who is placed in the country during the first few years of a new FETP to guide training and provide technical assistance. Since 1980, CDC has supported the launch of ≈45 FETPs with participants from ≈64 countries; numbers have increased since 2000 (Figure 1). Almost all of these FETPs continue to recruit and train epidemiologists, and many function independently of CDC funding. As of December 2016, there were 65 FETPs in 90 countries, and CDC was supporting 30 FETP-Advanced serving 49 countries (Figure 2); ≈3,900 field epidemiologists have graduated from these CDC-supported programs. 

FETPs traditionally have been 2-year programs that are based in ministries of health and that provide advanced field epidemiology training and service; however, shorter FETP models now exist. Participants (residents) in the FETPs-Advanced, usually ministry of health physicians and other professional staff, learn and practice epidemiologic skills while delivering essential epidemiologic services (training through service) to the ministry at the national or subnational level. FETP residents contribute to ministry missions by reviewing and analyzing surveillance data, detecting and responding to disease outbreaks and other public health emergencies, and conducting planned studies of public health priorities. They also develop skills to conduct public health research, improve communication of scientific findings, translate those findings into public health action, and contribute to the network of field epidemiologists locally and worldwide (*9*,*10*).

As a fundamental feature, FETPs use the learning-by-doing approach with mentored public health practice for >70% of program time (*4*,*11*). However, programs are tailored to suit the needs and conditions of individual countries and regions. For example, although the focus for most programs is national, for a few programs it is regional (e.g., Central America, French-speaking West Africa, central Asia) (*12*,*13*), and some national programs accept residents from smaller neighboring countries. Many FETPs partner with a university to provide a postgraduate degree to residents who successfully complete the field and academic requirements, and some offer medical board qualification in community medicine or epidemiology. Some programs have included a laboratory track (Field Epidemiology and Laboratory Training Program; FELTP) (*14*), a veterinary track, or both, and 1 has a parallel veterinary FETP for animal health. The Central America program addressed the need for improved surveillance and epidemiology practice at all levels of the public health system by developing a 3-tiered FETP training model (Basic/Frontline, Intermediate, and Advanced) to build capacity at each level (*12*,*15*). Each tier aims to improve competency of public health workers in the same 4 essential domains of field epidemiology—surveillance, field investigation and response, data collection and analysis, and scientific communication—but the expectations are tailored to the public health skills needed at that level. FETP-Frontline training for surveillance officers has been implemented throughout Africa, Latin America, and elsewhere in response to the Ebola epidemic in West Africa, the Zika virus threat in the Americas, and the adoption of the GHSA (a global initiative to strengthen capacity to prevent, detect, and respond to public health threats). As of the end of 2016, a total of 24 new Frontline programs had been established and 1,354 surveillance staff had been trained (*16*).

During the 1990s, directors of several FETPs and similar programs organized themselves into a global network to expand program reach and to ensure program quality. In 1997, the network was formalized as the Training Programs in Epidemiology and Public Health Interventions Network (TEPHINET) (*17*). TEPHINET now has 69 member programs in its global network. Over the years, as the number of FETPs has expanded, regional networks have been developed to support program implementation and strengthening. The African Field Epidemiology Network formed in 2005 (*18*), the Eastern Mediterranean Public Health Network (*19*) and the South East Asia Field Epidemiology and Technology Network in 2009, and the South American Field Epidemiology Training Programs Network and the Association of Southeast Asian Nations Plus Three Field Epidemiology Training Networks in 2011.

TEPHINET, along with its member programs and CDC, has recently developed and implemented an accreditation process for FETPs-Advanced (http://www.tephinet.org/accreditation). Accreditation was initiated in response to the increasing numbers of programs and the variations in their implementation. Its goal is to maintain and improve program quality (*20*,*21*). The process has received wide support from FETP program directors (D. Herrera, TEPHINET, pers. comm., 2017 Feb 28). The first 3 programs (EIS, Canadian Field Epidemiology Program, and the UK FETP) were accredited in 2016, and more programs have applied for accreditation in 2017.

## Outcomes and Effects of FETPs

The goal of FETPs is to develop competent field epidemiologists who can assume priority public health positions while strengthening countries’ outbreak response capacity, surveillance systems, and use of data to inform prevention and control measures for priority public health problems. The following examples demonstrate the value of a strong public health workforce and improved surveillance, outbreak response, and data use capacity for greatly enhancing national, regional, and global health security.

### Outbreak Investigations and Emergency Responses

**Figure 3:**
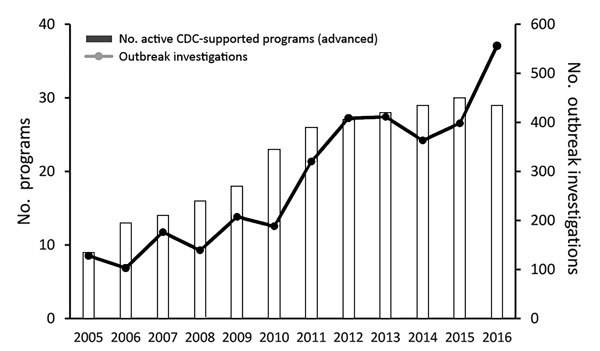
Outbreak investigations conducted by residents (participants) in US Centers for Disease Control and Prevention (CDC)–supported Field Epidemiology Training Programs, 2005–2016.

Since 2005, FETP residents have responded to ≈3,300 outbreaks (Figure 3). Although many of these outbreaks were local, the experience prepared FETP residents to handle problems of national and international concern.

During the recent Ebola epidemic in West Africa, ≈70 FETP residents and graduates from >9 African nations and Haiti participated in investigation and response activities. They served as epidemiologists, surveillance officers, contact tracing supervisors, and laboratorians in support of epidemic control (*22*) (L. Boulanger, CDC Ethiopia, pers. comm., 2017 Mar 23). In 2015, the residents and graduates of the Nigeria FELTP supported a contact tracing effort that prevented a major Ebola epidemic in that country, in contrast to the unchecked spread in neighboring countries without FETPs (*23*,*24*).

In February 2015, the 10 residents of the Uganda FETP were called to investigate an outbreak of a strange disease that had killed 1 person and sickened 24 more in Kampala, the capital of Uganda. Investigation by the residents uncovered a much more widespread outbreak of typhoid fever that had spread insidiously throughout Kampala. They identified the cause as contaminated water from uncontrolled underground sources (*25*). Guided by this epidemiologic investigation, international institutes and organizations from Uganda and elsewhere mounted a major coordinated response and contained the outbreak.

In May and June 2013, the India Epidemic Intelligence Service (India EIS), an FETP in India, investigated an outbreak of unexplained encephalopathy in which 133 children were hospitalized and 59 (44%) died. Similar outbreaks had been noticed since 1995, but multiple attempts to find a cause and control the disease had failed. The India EIS noted that many of the affected children were hypoglycemic, a characteristic of patients with ackee fruit encephalopathy. They also noted that litchi (also called lychee) fruit, a relative of the ackee, was commercially cultivated in the area. When the outbreak recurred in 2014, the India EIS demonstrated a strong epidemiologic association between encephalopathy and litchi consumption; laboratory testing confirmed the presence of the toxins methylenecyclopropylglycine and hypoglycin A in affected children and in litchi. Evidence-based recommendations were developed to prevent future seasonal outbreaks and associated deaths (*26*).

In 2007 in China, paraplegia suddenly developed in leukemia and lymphoma patients while they were receiving weekly intrathecal injections of drug. Without an identified cause, the intrathecal drugs were embargoed, thus limiting treatment availability. Investigation by epidemiologists and residents of the China FETP led to the identification of contamination with minute quantities of vincristine, a potent neurotoxin (*27*). These findings enabled correction of the problem and resumption of intrathecal drug production and use.

### Surveillance System Support

During their training, all FETP residents are expected to analyze, use, and improve surveillance data. Surveillance systems addressed by FETP residents include those for routinely reported notifiable diseases; specific diseases, such as HIV infection; and noncommunicable conditions such as maternal death, injury, and birth defects (*28*,*29*).

During the 2014 outbreak of Middle East respiratory syndrome (MERS) in Jeddah, Saudi Arabia, several graduates from the Saudi Arabia FETP were asked to strengthen the surveillance system for MERS. The graduates tackled numerous issues such as nonuse of the case definition for selection of laboratory testing, delayed laboratory reporting, and inconsistent case counts among sources. The FETP team redesigned the system to enable simultaneous real-time electronic reporting of suspected and confirmed cases to public health professionals who needed to take essential control and preventive actions on new cases. The system, now run by another FETP graduate, provides real-time data on MERS in Saudi Arabia and is used to populate the widely distributed, weekly Saudi MERS report that is redistributed by WHO (A. Alzahrani, King Faisal Specialist Hospital and Research Center, pers. comm., 2017 Feb 7).

FETPs also develop and support surveillance and response systems during mass gatherings for sporting, religious, and other events. During the Fédération Internationale de Football Association World Cup held in South Africa in 2010, FETP residents helped establish and run surveillance and response systems to protect the public health during these events. The 22 FETP residents supported the collation and analysis of data from the 9 provinces and participated in investigation of ≈20 suspect public health events (*30*) (L. Kuonza, South Africa FETP, pers. comm., 2017 Feb 2). Similarly, during religious mass gathering events in Saudi Arabia, Pakistan, Morocco, Iraq, and Jordan, FETP residents in those countries supported surveillance and other public health activities (*31*–*34*).

FETP-trained personnel also participate in surveillance activities during national disasters. In 2010, when floods covered 20% of Pakistan, FETP-trained officials were mobilized to help their provincial departments of health. They developed and maintained surveillance and responded to outbreaks in the camps of displaced populations. This workforce provided vital public health services, including planning; coordination; data collection, analysis, and interpretation; emergency preparedness and response; and outbreak investigations in multiple districts (*35*).

FETPs have also strengthened laboratory surveillance. The South Caucasus FELTP expanded the existing anthrax surveillance to include poxviruses, leading to improved diagnosis and control for anthrax and identification of a novel poxvirus (*36*).

### Control and Prevention of Priority Public Health Problems

FETPs play critical roles in addressing priority public health problems in countries, often with the collaboration and support of international initiatives such as the US President’s Emergency Plan for AIDS Relief (*37*,*38*), the President’s Malaria Initiative (*39*,*40*), and the Global Polio Eradication Initiative (*41*,*42*). This approach ensures that residents’ work supports national and global priorities while the residents practice applying epidemiologic methods to these programs and providing public health service.

As an example, FETP residents have broadly supported polio elimination in Nigeria and Pakistan through National Stop Transmission of Polio (N-STOP) programs. FETP Pakistan designed the first N-STOP program to meet the need for local public health staff to fight polio in that country. The North Waziristan area, a highly security-compromised area, reported 20% of all global cases of polio in 2014. Despite ongoing military operations and human displacement, FETP Pakistan placed 2 residents as N-STOP officers in the North Waziristan area and the adjoining South Waziristan area. The residents, working under difficult and hazardous conditions, rebuilt the infrastructure for surveillance and polio eradication activities and persuaded other staff to return. The transmission of polio virus was interrupted in the North Waziristan area (cases decreased from 70 in 2014 to 0 in 2016) and substantially reduced in the South Waziristan area (cases decreased from 24 in 2014 to 2 in 2016) (*42*).

In Nigeria, the N-STOP program has developed innovative strategies to address polio eradication challenges. One N-STOP initiative focused on locating and vaccinating children <5 years of age in remote, nomadic, scattered, and border populations in northern Nigeria where low polio vaccination coverage probably contributed to ongoing transmission of wild polio virus. During August 2012­­–April 2013, N-STOP conducted field outreach activities that enumerated ≈40,000 remote settlements, including 4,613 settlements never visited by vaccination teams during previous polio supplemental immunization activities (*41*,*43*).

## Graduates

Training competent field epidemiologists for a country builds long-term capacity only if the country uses FETP graduates in appropriate positions of public health responsibility. Some countries have developed specific positions for graduates, such as provincial epidemiologist. Other countries have modified the requirements for certain positions to include FETP certification. Overall, most FETP graduates are retained within their country’s public health systems, and many rise to positions of public health responsibility. We estimate that ≈80% of recent graduates continue to work for the national ministry of health or equivalent. In many countries, this figure approaches 100%. Graduates have served as permanent secretaries for health; ministers of health; and program directors for epidemiology, surveillance, and specific disease control programs. Others have held responsible positions with WHO (e.g., national professional officers) and nongovernmental public health–associated organizations.

A valued role for graduates is leadership within the FETP itself. The national FETP director and other technical staff are usually FETP graduates. Graduates serving in national and other public health positions are specifically groomed to serve as mentors for the residents during their field placements. Experienced graduates have also been hired to serve as resident advisors of newly developed FETPs in other countries.

## Building Institutionalized and Sustainable FETPs

Most FETPs were initiated with financial and technical support from external donors and partners (*3*,*4*). The costs for developing programs vary widely and, among other considerations, depend on the size, model, and partners involved (*10*). To ensure their continuity and long-term contribution toward strengthening public health, the programs are anchored within the ministries of health or other public health institutions. This national ownership ensures that the FETPs contribute to tangible and relevant delivery of essential epidemiologic services from the outset.

Recognizing FETPs as valuable for addressing national health priorities has helped to institutionalize and sustain FETPs (*11*,*44*). Many programs have been operating independently for years and have become national resources for disease surveillance, public health emergency response, and priority public health disease prevention and control programs (*45*,*46*).

Of the 19 programs that were established during 1980–2000 with CDC engagement, 17 continue to produce graduates and provide service. The principal elements for program institutionalization and sustainability include establishment of an organizational structure and institutional ownership within the ministry of health or other public health institution, national leadership from FETP graduates, focus on priority- and science-based training, communication of findings and recommendations to the public health leadership, assurance of a recognized career path for graduates, and continued engagement between graduates and the FETP (*20*). CDC works with programs to support these elements and to help ensure their long-term success.

## Challenges

Despite progress in building sustainable institutionalized programs, several challenges remain. New FETPs commonly struggle to identify sufficient numbers of qualified epidemiologists to serve as mentors until graduates can become mentors at least 2 years later. Ministries of health commonly wrestle with the challenge to develop and maintain appropriate career paths for FETP graduates. In the absence of appropriate available positions, graduates often resume their pretraining roles, which probably underutilize their new epidemiologic skills. Committed ministries of health have had varying levels of success in addressing this problem, depending on the structure and limitations of their human resources systems (*47*,*48*). A final challenge is that uncertain political support within the health system, funding limitations in the face of competing priorities, and weaknesses in the healthcare infrastructure can threaten support for FETPs and prevent establishment of a sufficient institutional framework to ensure long-term survival. CDC works with programs to identify and engage numerous disease initiatives and multisectoral global health activities to develop new partnerships to support programs as they develop (*49*). To highlight the contribution of FETPs and promote their sustainability in countries around the world, continuous advocacy is essential.

## Conclusions

In this age of globalization and the emergence of new and resurgent communicable diseases (e.g., Ebola, Zika, MERS) and the increasing global effects of known diseases (e.g., yellow fever, dengue fever), qualified field epidemiologists are needed more than ever. There is a critical need for good epidemiologic science in all countries to support prevention and control programs for communicable and noncommunicable diseases, injuries, and environmental hazards. The adoption of the IHR 2005 standards and the development of the GHSA have made clear that every country needs at least a minimum capacity in field epidemiology to rapidly detect, respond to, and control public health emergencies and thereby keep its population safe, protect other countries from the spread of illness, and ensure global health security. The development of FETPs across the globe is recognized as being critical for meeting that need and therefore for enhancing global health security (*50*). It will be crucial to maintain and continue to improve the quality and reach of FETPs in countries through expanding the number of countries with access to these programs and expanding the tiered training within countries. The global public health community, working together with international partners and the global network of FETPs, can be instrumental in building on the strengths of the existing programs to broaden the beneficial effects of these critical capacity-building efforts.
